# Clinical Implications of the Association between Respiratory and Gastrointestinal Disorders in Migraine and Non-Migraine Headache Patients

**DOI:** 10.3390/jcm12103434

**Published:** 2023-05-12

**Authors:** Jong-Ho Kim, Yeonkyeong Lee, Young-Suk Kwon, Jong-Hee Sohn

**Affiliations:** 1 Department of Anesthesiology and Pain Medicine, Chuncheon Sacred Heart Hospital, Hallym University College of Medicine, Chuncheon 24253, Republic of Korea; poik99@hallym.or.kr (J.-H.K.); gettys@hallym.or.kr (Y.-S.K.); 2 Institute of New Frontier Research Team, College of Medicine, Hallym University, Chuncheon 24252, Republic of Korea; leeyk10047@naver.com; 3 Department of Neurology, Chuncheon Sacred Heart Hospital, Hallym University College of Medicine, Chuncheon 24253, Republic of Korea

**Keywords:** migraine, respiratory disorders, headache, gastrointestinal disorders, asthma

## Abstract

Headaches, particularly migraine, are associated with gastrointestinal (GI) disorders. In addition to the gut–brain axis, the lung–brain axis is suspected to be involved in the relationship between pulmonary microbes and brain disorders. Therefore, we investigated possible associations of migraine and non-migraine headaches (nMH) with respiratory and GI disorders using the clinical data warehouse over 11 years. We compared data regarding GI and respiratory disorders, including asthma, bronchitis, and COPD, among patients with migraine, patients with nMH, and controls. In total, 22,444 patients with migraine, 117,956 patients with nMH, and 289,785 controls were identified. After adjustment for covariates and propensity score matching, the odds ratios (ORs) for asthma (1.35), gastroesophageal reflux disorder (1.55), gastritis (1.90), functional GI disorder (1.35), and irritable bowel syndrome (1.76) were significantly higher in patients with migraine than in controls (*p* = 0.000). The ORs for asthma (1.16) and bronchitis (1.33) were also significantly higher in patients with nMH than in controls (*p* = 0.0002). When the migraine group was compared with the nMH group, only the OR for GI disorders was statistically significant. Our findings suggest that migraine and nMH are associated with increased risks of GI and respiratory disorders.

## 1. Introduction

Headaches, particularly migraine, are associated with various gastrointestinal (GI) symptoms and several GI disorders. The gut–brain axis facilitates bidirectional communication between the brain and gut through neural, endocrine, and immune pathways [1,2,3].

There may be a connection between headache and the gut. GI symptoms, such as nausea and vomiting, often occur with migraine attacks. Previous studies have reported that GI disorders associated with primary headaches include dyspepsia, gastroesophageal reflux disease (GERD), constipation, functional abdominal pain, inflammatory bowel syndrome (IBS), inflammatory bowel disorders, celiac disease, and *Helicobacter pylori* infection [4]. In particular, an increased frequency of GI disorders has been demonstrated in patients with migraine compared with the general population. *H. pylori* infection, IBS, gastroparesis, hepatobiliary disorders, celiac disease, and changes in the microbiota have been linked with the occurrence of migraine, although the precise mechanisms and pathways related to the gut–brain axis in patients with migraine need to be fully elucidated [5].

In addition to the gut–brain axis, the lung–brain axis is suspected to be involved in the relationship between the central nervous system and lungs through neuroanatomical, endocrine, immune, metabolic, microbial, and gas-mediated pathways [6]. A recent report demonstrated the existence of the lung–brain axis, through which the lung microbiome regulates the magnitude of autoimmune inflammation in the brain [7].

The results of epidemiological studies suggest the presence of a communication network between the central nervous system and lungs. Previous studies have demonstrated that patients with chronic obstructive pulmonary disease (COPD) have an increased prevalence and incidence of ischemic or hemorrhagic stroke [8,9]. A recent study demonstrated that COPD is associated with cerebrovascular disease related to the presence of white matter lesions [10]. Several experimental and clinical studies have demonstrated an association between lung injury and multiple sclerosis or traumatic brain injury [11,12,13]. Furthermore, the results of recent studies have suggested that the pulmonary microbiome regulates the immune reactivity of central nervous tissue and influences the development of autoimmune or neurodegenerative disease [14,15].

Respiratory disorders, including asthma, bronchitis, and COPD, are significantly more common in individuals with chronic migraine [16]. A meta-analysis of observational studies revealed an increased prevalence and incidence of migraine among patients with asthma [10]. Similarly, a systematic review showed that migraine is associated with a significantly increased risk of asthma [17]. The findings in a previous study suggest that cardiac and pulmonary diseases affect the cranial final common pathway responsible for the onset of migraine attacks. This hypothesis was based on data linking migraine with right-to-left shunts and by analogy with etiologies of decompression illness [18].

However, compared to the known link between migraine and GI disorders, the association between primary headaches, including migraine and non-migraine headaches (nMH), and respiratory disorders, such as asthma, bronchitis, and COPD, remains unclear. Additionally, whether there is a difference in the association of GI and respiratory disorders between migraine and nMH patients is unknown. A better understanding of the relationship between common primary headache and respiratory or GI disorders is important, as it may have therapeutic consequences. Therefore, we investigated the association between primary headaches (migraine or nMH) and respiratory and GI disorders using data from the smart clinical data warehouse (CDW) over 11 years.

## 2. Materials and Methods

### 2.1. Subjects

**Figure 1:**
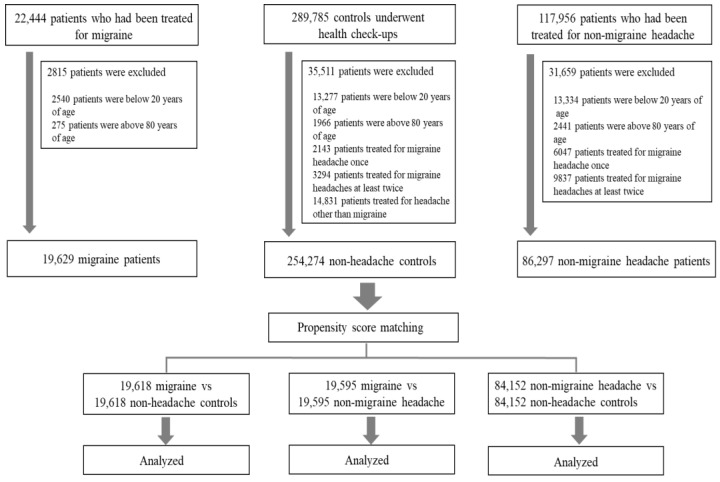
Flow chart of the enrollment process.

We analyzed clinical data from the smart CDW of the Hallym University Medical Center between January 2012 and November 2022. The smart CDW is based on the QlikView Elite Solution (Qlik, Lund, Sweden) and is used at all five Hallym University Medical Center hospitals. It offers text data and integrated analysis of fixed data from electronic medical records. Patients with migraine were eligible for inclusion if they met the following criteria: age 20–80 years; diagnosis of migraine after assessment by board-certified neurologists; presence of International Classification of Diseases tenth revision (ICD-10) code G43 in medical records; and a history of more than two outpatient visits or at least one admission to the neurology department. Patients with nMH were eligible if they were aged 20–80 years and had two diagnosis ICD-10 codes (G44 and R51) in the database, indicating tension-type headache (TTH) or non-specific headaches. Subjects with a history of more than one visit for treatment of migraine were excluded from the nMH group. The control group included patients aged 20–80 years who had undergone general health checkups at a health promotion center. Patients with a history of headaches or migraine were assessed using a basic questionnaire completed before their health checkups; patients who visited our medical center for the treatment of headaches or migraine were excluded. The enrollment process is presented in Figure 1. In total, 22,444 patients with migraine, 117,956 patients with nMH, and 289,785 controls were enrolled. After excluding subjects based on the exclusion criteria, 19,629 migraine, 86,297 nMH, and 254,274 non-headache controls were included in the study (Figure 1). The Clinical Research Ethics Committee of Chuncheon Sacred Heart Hospital, Hallym University, approved the study protocol (IRB no. 2022-10-010). Because only de-identified data were used in this study, the requirement for informed consent was waived by the review board.

### 2.2. Migraine or nMH, Respiratory Disorders, and Covariates

We compared the prevalence of GI disorders (such as GERD, gastritis, functional GI disorder (FGID), and IBS) and respiratory disorders (such as asthma, bronchitis, and COPD) in patients with migraine and patients with nMH to the prevalence in controls. Definite cases were selected based on diagnosis codes in the database and a history of more than two outpatient visits or at least one admission for each diagnosis. Covariates included age, sex, and various comorbidities, defined using relevant ICD-10 codes in the database. These comorbidities included angina (I20, I24, and I251), atrial fibrillation (I480–482 and I489), anxiety disorder (F41), cerebrovascular diseases (G45–46 and I60–69), chronic hepatitis (B18, I85, and K70–74), depression (F31–34, F412, and F432), diabetes mellitus (E10–14), dyslipidemia (E78), heart disease (I05–09, I21–23, I30–47, and I49–52), hypertension (I10–15), menopause (M800, M010, N924, and N95), renal failure (N03 and N18–17), and sleep disorders (F51, G258, and G47).

### 2.3. Statistical Analysis

Categorical data are presented as frequencies and percentages, whereas continuous data are presented as means and standard deviations. The chi-squared test was used to analyze categorical data, and *t*-tests were performed to compare continuous data among patients with migraine, patients with nMH, and controls. Because of the inability to randomize patients based on migraine or nMH status, we adjusted for covariates and selection biases using propensity scores. Propensity score matching (PSM) was performed among patients with migraine, patients with nMH, and controls using Python (version 3.7, https://www.anaconda.com, accessed on 25 March 2023; Anaconda Inc., Austin, TX, USA) and Pymatch (version 0.3.4; https://github.com/benmiroglio/pymatch, accessed on 25 March 2023). Propensity scores ranged from 0.07 to 0.87; all matched cases had scores within 0.0001 of each other at a matching ratio of 1:1. This process resulted in 19,618 matched pairs of migraine patients and normal controls, 84,152 matched pairs of nMH patients and normal controls, and 19,595 matched pairs of migraine patients and nMH patients. Odds ratios (ORs) with 95% confidence intervals (CIs) were calculated using logistic regression to measure the associations between exposure and outcome for respiratory disorders (such as asthma, bronchitis, and COPD) and GI disorders (such as GERD, gastritis, FGID, and IBS) compared with their respective controls. After PSM, adjusted ORs were calculated for each disorder among the three groups using covariates and propensity scores [19,20]. The Bonferroni correction was applied to correct for multiple testing, and *p*-values < 0.002 were considered significant. This value was derived by dividing the *p*-value threshold of 0.05 by the number of tests performed, i.e., 21 (seven outcome variables tested in three groups). All *p*-values were two sided. SPSS software (version 24.0; IBM Corp., Armonk, NY, USA) was used for statistical analyses.

## 3. Results

### 3.1. Subject Characteristics

Among the 19,629 enrolled patients with migraine, 14,357 were women (73.1%), with a mean age of 44.5 ± 14.5 years. In total, 86,297 patients with nMH and 254,274 controls were identified from the same database. Additionally, 56,760 (58.8%) patients with nMH and 124,936 (49.1%) controls were women; their mean ages were 49.2 ± 14.6 and 45.7 ± 15.0 years, respectively. After PSM, all absolute standardized differences between migraine and control groups were < 0.1 (Table 1). Similarly, no significant differences were observed for any variable between the nMH and control groups (Table 2). In a comparative analysis of migraine and nMH groups, no significant differences were observed for any variable after PSM (Table 3).

### 3.2. ORs for Respiratory and GI Disorders in Patients with Migraine and Patients with nMH

**Figure 2:**
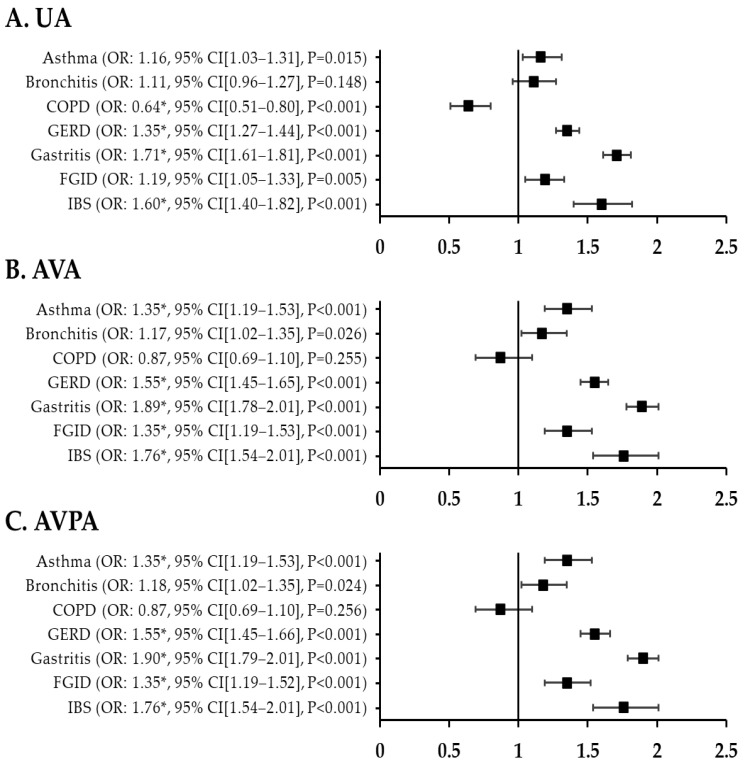
ORs for respiratory and GI disorders in migraine patients compared with controls after PSM. (**A**) unadjusted ORs (**B**) all variables adjusted ORs (**C**) all variables plus propensity score adjusted ORs * p < 0.002. UA, unadjusted; AVA, all variables adjusted; AVPA, all variables plus propensity score adjusted; GI, gastrointestinal; COPD, chronic obstructive pulmonary disease; GERD, gastroesophageal reflux disease; FGID, functional gastrointestinal disorder; IBS, irritable bowel syndrome; OR, odds ratio; CI, confidence interval.

**Figure 3:**
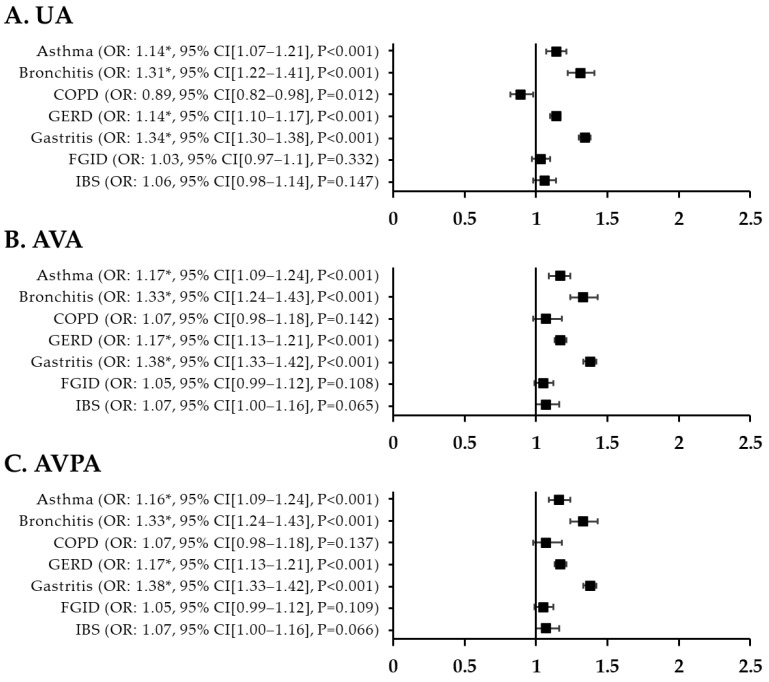
T. ORs for respiratory and GI disorders in nMH patients compared with controls after PSM. (**A**) unadjusted ORs (**B**) all variables adjusted ORs (**C**) all variables plus propensity score adjusted ORs * p < 0.002. UA, unadjusted; AVA, all variables adjusted; AVPA, all variables plus propensity-score adjusted; nMH, non-migraine headache; GI, gastrointestinal; COPD, chronic obstructive pulmonary disease; GERD, gastroesophageal reflux disease; FGID, functional gastrointestinal disorder; IBS, irritable bowel syndrome; OR, odds ratio; CI, confidence interval.

The adjusted ORs for respiratory disorder in patients with migraine versus controls were 1.35 (95% CI, 1.19–1.53; *p* < 0.001) for asthma, 1.17 (95% CI, 1.02–1.35; *p* = 0.026) for bronchitis, and 0.87 (95% CI, 0.69–1.10; *p* = 0.025) for COPD. The fully adjusted OR for asthma in patients with migraine was also high at 1.35 (95% CI, 1.19–1.53; *p* < 0.001). After adjustment for all variables and PSM, the fully adjusted ORs were higher for GI disorders than for respiratory disorders in patients with migraine: 1.55 (95% CI, 1.45–1.66) for GERD, 1.90 (95% CI, 1.79–2.01) for gastritis, 1.35 (95% CI, 1.19–1.52) for FGID, and 1.76 (95% CI, 1.54–2.01) for IBS (*p* < 0.001) (Figure 2). Figure 3 shows the ORs for respiratory and GI disorders in nMH patients versus controls. The fully adjusted ORs were 1.16 (95% CI, 1.09–1.24) for asthma, 1.33 (95% CI, 1.24–1.43) for bronchitis, 1.17 (95% CI, 1.13–1.21) for GERD, and 1.38 (95% CI, 1.33–1.42) for gastritis (*p* < 0.001) (Figure 3). Additionally, we have added a table presenting the prevalence of the outcome variables among the patients with migraine, patients with nMH, and controls (Table A1).

### 3.3. Differences in Respiratory and GI Disorders between Patients with Migraine and Patients with nMH

**Figure 4:**
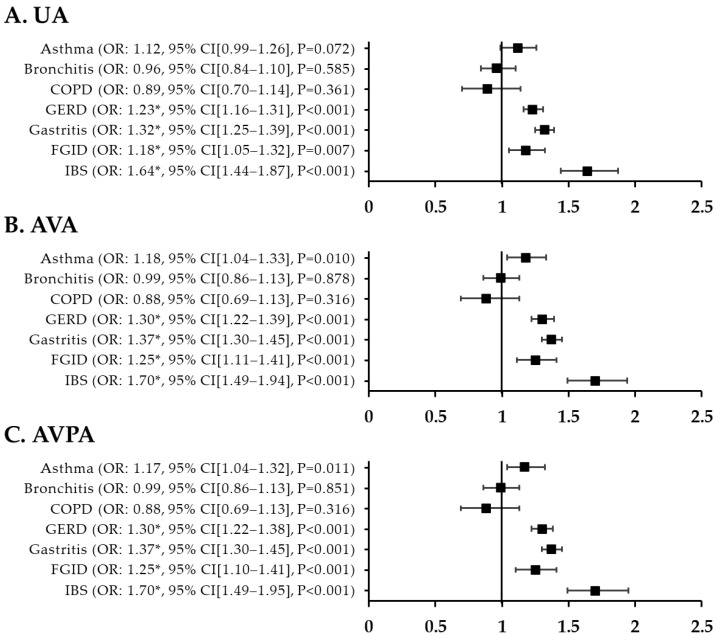
ORs for respiratory and GI disorders in migraine patients compared with nMH patients after PSM. (**A**) unadjusted ORs (**B**) all variables adjusted ORs (**C**) all variables plus propensity score adjusted ORs * *p* < 0.002. UA, unadjusted; AVA, all variables adjusted; AVPA, all variables plus propensity-score adjusted; nMH, non-migraine headache; GI, gastrointestinal; COPD, chronic obstructive pulmonary disease; GERD, gastroesophageal reflux disease; FGID, functional gastrointestinal disorder; IBS, irritable bowel syndrome; OR, odds ratio; CI, confidence interval.

In a comparative analysis of patients with migraine and patients with nMH, the fully adjusted ORs for GERD, gastritis, FGID, and IBS were all significantly higher in migraine patients than in nMH patients. The ORs were 1.30 (95% CI, 1.22–1.38; *p* < 0.001) for GERD, 1.37 (95% CI, 1.30–1.45; *p* < 0.001) for gastritis, 1.25 (95% CI, 1.10–1.41; *p* < 0.001) for FGID, and 1.70 (95% CI, 1.49–1.95; *p* < 0.001) for IBS. In contrast, among respiratory disorders, there were no statistically significant differences between migraine and nMH patients (Figure 4).

## 4. Discussion

In this study, we compared clinical data regarding respiratory and GI disorders among patients with migraine, patients with nMH, and controls. After adjusting for covariates and PSM, the ORs for asthma and all GI disorders, including GERD, gastritis, FGID, and IBS, were significantly higher in patients with migraine than in controls. The ORs for asthma and bronchitis were significantly higher in patients with nMH than in controls. However, when comparing the migraine and nMH groups, there were no statistically significant differences in respiratory disorders, but the ORs for all GI disorders, including GERD, gastritis, FGID, and IBS, were significant. Our findings suggest that migraine and nMH patients have an increased risk of GI and respiratory disorders.

Previous epidemiological studies revealed a high prevalence of asthma in patients with migraine [21,22]; migraine is also significantly more common in patients with asthma, particularly adolescents [23,24,25]. Migraine and asthma exhibit a bidirectional association, such that each condition is reciprocally associated with the other [26,27]. Additionally, asthma increases the risk of chronic migraine 1 year later among individuals with episodic migraine, potentially influencing the clinical course of migraine [28]. Migraine headaches may also be associated with poor asthma control [29]. Migraine and asthma are chronic disorders characterized by recurrent episodic attacks; they share underlying pathophysiological mechanisms involving inflammation and neurological processes. Both conditions are influenced by hormonal imbalances and environmental triggering factors [30,31,32]. However, only a few studies have investigated associations between migraine and respiratory disorders other than asthma or between respiratory disorders and primary headaches other than migraine.

In a comparative study of chronic and episodic migraine groups based on a large population sample, respiratory disorders (e.g., asthma, bronchitis, and COPD) were significantly more common in individuals with chronic migraine [16]. Among 288 outpatients with asthma diagnosed through early or late reversibility tests, 60.4% reported headaches, and 32.6% met the International Headache Society criteria for migraine [24]. Our study revealed an association between migraine and asthma that was similar to the findings in previous studies. Additionally, the ORs for asthma and bronchitis were significantly higher in the nMH group than in the control group. The nMH group in our study included patients with TTH or non-specific headache codes; it excluded patients with all types of migraines. Thus, our findings suggest an association between primary headaches and respiratory disorders.

A previous large, population-based, cross-sectional study showed that migraine and nMH were 1.5-fold more likely among patients with asthma, asthma-related symptoms, hay fever, and chronic bronchitis than among patients without these conditions [33]. Previous studies demonstrated a plausible relationship between primary headaches and immunological or autoimmune disorders [34,35]; immunological events are potentially involved in the pathophysiology of primary headaches. The prevalence of headaches is higher in patients with immunological or autoimmune disorders than in the general population, suggesting that headaches constitute a risk factor or clinical manifestation of these conditions [35]. Cytokine profiles of cluster headaches and TTH patients suggest immunological dysregulation in the pathophysiology of primary headaches, but the corresponding evidence is weaker than the evidence for migraine [34]. Although the presence of a causal relationship is uncertain, our results suggest that comorbid conditions (e.g., allergies and respiratory disease) should be considered in patients with frequent primary headaches.

Our results showed that the ORs for respiratory and GI disorders (e.g., asthma, GERD, gastritis, FGID, and IBS) were significantly higher in patients with migraine than in controls. Compared with the control group, the nMH group also had significantly greater risks of respiratory and GI disorders, although the ORs for these conditions, including asthma, GERD and gastritis, were lower in the nMH group than in the migraine group. However, only the ORs for GI disorders were statistically significant in the comparison between the migraine and nMH groups. The brain has many connections with various organs. Headaches, particularly migraine, are associated with GI disorders and are often accompanied by various GI symptoms [1,2,3,5,36,37,38,39]. While nearly all previous observational studies focused on migraine, some studies investigated the relationship between GI symptoms and headache, including migraine. There was a statistically significant association between TTH and the prevalence of GI disorders [37,38]. All GI complaints were as common among subjects with nMH as in migraineurs, and the strength of the association between headache and GI complaints increased with increasing headache frequency [39]. Additionally, some studies reported an association between primary headache and GI disorders and demonstrated improvement of headache following treatment of the accompanying GI disorder [40,41,42,43,44]. Current evidence shows that the gut–brain axis may affect migraine, although the mechanism explaining this interaction is not entirely clear. Emerging evidence suggests that the gut microbiota is crucial in the pathogenesis of migraine [2]. Studies using animal models and human studies have demonstrated that the gut microbiome is altered in migraine sufferers compared to healthy controls [45]. Recent evidence suggests that the gut microbiota may also play a critical role in many other types of chronic pain, including headache [46]. Therefore, further epidemiological, clinical, and pathophysiological evidence is needed to identify associations between primary headaches other than migraine and the gut microbiome. In addition to the gut microbiome, the lung microbiome regulates the immune reactivity of the brain [6]. Experimental reports have described immunological findings in patients with migraine and other primary headaches [34]. Thus, more research is needed to determine the association between primary headache and the lung microbiome.

This study had some limitations. First, it was a retrospective study that used clinical data from individuals who visited a university medical center with five hospitals. Thus, it is difficult to generalize the results to the general population; there was also potential for selection bias and unmeasured confounding variables that were not considered. Second, patients were included according to diagnosis codes in the CDW, but detailed clinical information regarding primary headache or respiratory disease characteristics was not collected. Additionally, various psychiatric comorbidities are common in patients with primary headache. Of the psychiatric comorbidities, only depression and anxiety disorders, which are the most prevalent in headache patients, were selected and analyzed as covariates in this study. However, other psychiatric comorbidities should also be considered. To increase the accuracy of patient inclusion, data were collected for patients who had a history of at least two outpatient treatments or at least one hospitalization for each diagnostic code of migraine, GI, and respiratory disorders. Future prospective, population-based studies are needed to investigate the association between primary headaches and respiratory and GI disorders.

## 5. Conclusions

In addition to GI disorders, our findings indicate that migraine and nMH are associated with increased risks of respiratory disorders, such as asthma and bronchitis. However, comparison of the migraine and nMH groups revealed that there were no statistically significant differences in respiratory disorders, but significant differences were shown in all GI disorders. Our findings suggest that migraine and nMH are associated with increased risks of GI and respiratory disorders. Future prospective studies are needed to provide further evidence and fully investigate the associations between primary headache disorders and respiratory and GI disorders.

## Figures and Tables

**Table 1:** Characteristics of the migraine and control groups before and afterpropensity score matching.

	Before Matching			After Matching		
	Migraine	Control	ASD	Migraine	Control	ASD
	*n* = 19,629	*n* = 254,274		*n* = 19,618	*n* = 19,618	
Female sex	14,357 (73.1%)	124,936 (49.1%)	0.542	14,346 (73.1%)	14,231 (72.5%)	0.013
Age (y)	44.5 ± 14.5	45.7 ± 15.0	0.079	44.5 ± 14.5	46.0 ± 15.3	0.106
DM	976 (5.0%)	13,150 (5.2%)	0.009	976 (5.0%)	1030 (5.3%)	0.013
HTN	2274 (11.6%)	20,391 (8.0%)	0.111	2272 (11.6%)	2437 (12.4%)	0.026
Dyslipidemia	1847 (9.4%)	21,278 (8.4%)	0.036	1846 (9.4%)	1997 (10.2%)	0.026
Angina	1110 (5.7%)	8161 (3.2%)	0.106	1109 (5.7%)	1164 (5.9%)	0.012
AF	150 (0.8%)	2231 (0.9%)	0.013	150 (0.8%)	177 (0.9%)	0.016
Heart disease	882 (4.5%)	6762 (2.7%)	0.089	881 (4.5%)	954 (4.9%)	0.018
CVD	2779 (14.2%)	9601 (3.8%)	0.298	2768 (14.1%)	2989 (15.2%)	0.032
Renal failure	263 (1.3%)	3145 (1.2%)	0.009	263 (1.3%)	312 (1.6%)	0.022
Chronic hepatitis	476 (2.4%)	10,099 (4.0%)	0.101	475 (2.4%)	475 (2.4%)	<0.001
Anxiety	818 (4.2%)	2217 (0.9%)	0.165	813 (4.1%)	788 (4.0%)	0.006
Depression	2315 (11.8%)	4896 (1.9%)	0.306	2304 (11.7%)	2302 (11.7%)	<0.001
Sleep disorder	1553 (7.9%)	4227 (1.7%)	0.232	1542 (7.9%)	1495 (7.6%)	0.009
Menopause	708 (3.6%)	6386 (2.5%)	0.059	707 (3.6%)	760 (3.9%)	0.014

ASD, absolute standardized difference; DM, diabetes mellitus; HTN, hypertension; AF, atrial fibrillation; CVD, cerebrovascular disease.

**Table 2:** Characteristics of the nMH and control groups before and after propensity score matching.

	Before Matching			After Matching		
	nMH	Control	ASD	nMH	Control	ASD
	*n* = 86,297	*n* = 254,274		*n* = 84,152	*n* = 84,152	
Female sex	50,760 (58.8%)	124,936 (49.1%)	0.197	49,071 (58.3%)	47,979 (57.0%)	0.026
Age (y)	49.2 ± 14.6	45.7 ± 15.0	0.242	49.0 ± 14.7	49.0 ± 15.2	<0.001
DM	5248 (6.1%)	13,150 (5.2%)	0.038	5089 (6.0%)	5397 (6.4%)	0.015
HTN	10,978 (12.7%)	20,391 (8.0%)	0.141	10,468 (12.4%)	10,648 (12.7%)	0.006
Dyslipidemia	8217 (9.5%)	21,278 (8.4%)	0.039	7970 (9.5%)	8388 (10.0%)	0.017
Angina	5445 (6.3%)	8161 (3.2%)	0.128	5209 (6.2%)	5219 (6.2%)	<0.001
AF	1093 (1.3%)	2231 (0.9%)	0.035	1054 (1.3%)	1092 (1.3%)	0.004
Heart disease	4029 (4.7%)	6762 (2.7%)	0.095	3876 (4.6%)	3826 (4.5%)	0.003
CVD	11,834 (13.7%)	9601 (3.8%)	0.289	9939 (11.8%)	9467 (11.2%)	0.016
Renal failure	1407 (1.6%)	3145 (1.2%)	0.031	1382 (1.6%)	1467 (1.7%)	0.008
Chronic hepatitis	2767 (3.2%)	10,099 (4.0%)	0.043	2744 (3.3%)	2922 (3.5%)	0.012
Anxiety	2973 (3.4%)	2217 (0.9%)	0.141	2631 (3.1%)	2138 (2.5%)	0.032
Depression	5145 (6.0%)	4896 (1.9%)	0.170	4775 (5.7%)	4356 (5.2%)	0.021
Sleep disorder	3546 (4.1%)	4227 (1.7%)	0.123	3334 (4.0%)	3159 (3.8%)	0.010
Menopause	2864 (3.3%)	6386 (2.5%)	0.045	2794 (3.3%)	2724 (3.2%)	0.005

nMH, non-migraine headache; ASD, absolute standardized difference; DM, diabetes mellitus; HTN, hypertension; AF, atrial fibrillation; CVD, cerebrovascular disease.

**Table 3:** Characteristics of the migraine and nMH groups before and after propensity score matching.

	Before Matching			After Matching		
	Migraine	nMH	ASD	Migraine	nMH	ASD
	*n* = 19,629	*n* = 86,297		*n* = 19,595	*n* = 19,595	
Female sex	14,357 (73.1%)	50,760 (58.8%)	0.323	14,323 (73.1%)	14,208 (72.5%)	0.013
Age	44.5 ± 14.5	49.2 ± 14.6	0.323	44.5 ± 14.5	44.8 ± 14.5	0.018
DM	976 (5.0%)	5248 (6.1%)	0.051	974 (5.0%)	1039 (5.3%)	0.015
HTN	2274 (11.6%)	10,978 (12.7%)	0.036	2269 (11.6%)	2319 (11.8%)	0.008
Dyslipidemia	1847 (9.4%)	8217 (9.5%)	0.004	1841 (9.4%)	1870 (9.5%)	0.005
Angina	1110 (5.7%)	5445 (6.3%)	0.028	1108 (5.7%)	1157 (5.9%)	0.011
AF	150 (0.8%)	1093 (1.3%)	0.058	150 (0.8%)	165 (0.8%)	0.009
Heart disease	882 (4.5%)	4029 (4.7%)	0.008	878 (4.5%)	855 (4.4%)	0.006
CVD	2779 (14.2%)	11,834 (13.7%)	0.013	2772 (14.1%)	2803 (14.3%)	0.005
Renal failure	263 (1.3%)	1407 (1.6%)	0.025	262 (1.3%)	307 (1.6%)	0.020
Chronic hepatitis	476 (2.4%)	2767 (3.2%)	0.051	476 (2.4%)	480 (2.4%)	0.001
Anxiety	818 (4.2%)	2973 (3.4%)	0.036	811 (4.1%)	846 (4.3%)	0.009
Depression	2315 (11.8%)	5145 (6.0%)	0.181	2281 (11.6%)	2304 (11.8%)	0.004
Sleep disorder	1553 (7.9%)	3546 (4.1%)	0.141	1519 (7.8%)	1533 (7.8%)	0.003
Menopause	708 (3.6%)	2864 (3.3%)	0.015	708 (3.6%)	690 (3.5%)	0.005

nMH, non-migraine headache; ASD, absolute standardized difference; DM, diabetes mellitus; HTN, hypertension; AF, atrial fibrillation; CVD, cerebrovascular disease.

## Data Availability

Not applicable.

## Appendix A

**Table A1:** Outcome variables in the migraine, nMH, and control groups.

	Before Matching			After Matching					
	Migraine	Controls	nMH	Migraine	Controls	nMH	Controls	Migraine	nMH
	*n* = 19,629	*n* = 254,274	*n* = 86,297	*n* = 19,618	*n* = 19,618	*n* = 84,152	*n* = 84,152	*n* = 19,595	*n* = 19,595
Asthma	585 (3.0%)	4051 (1.6%)	2313 (2.7%)	584 (3.0%)	505 (2.6%)	2233 (2.7%)	1972 (2.3%)	582 (3.0%)	523 (2.7%)
Bronchitis	431 (2.2%)	3050 (1.2%)	1844 (2.1%)	430 (2.2%)	389 (2.0%)	1778 (2.1%)	1360 (1.6%)	430 (2.2%)	446 (2.3%)
COPD	129 (0.7%)	2179 (0.9%)	984 (1.1%)	129 (0.7%)	200 (1.0%)	941 (1.1%)	1052 (1.3%)	128 (0.7%)	143 (0.7%)
GERD	2676 (13.6%)	19,015 (7.5%)	9518 (11.0%)	2671 (13.6%)	2050 (10.4%)	9177 (10.9%)	8184 (9.7%)	2665 (13.6%)	2217 (11.3%)
Gastritis	3600 (18.3%)	21,424 (8.4%)	12,038 (13.9%)	3596 (18.3%)	2279 (11.6%)	11,602 (13.8%)	8976 (10.7%)	3591 (18.3%)	2845 (14.5%)
FGID	619 (3.2%)	4302 (1.7%)	2140 (2.5%)	616 (3.1%)	522 (2.7%)	2059 (2.4%)	1998 (2.4%)	615 (3.1%)	525 (2.7%)
IBS	584 (3.0%)	3227 (1.3%)	1534 (1.8%)	584 (3.0%)	370 (1.9%)	1471 (1.7%)	1394 (1.7%)	579 (3.0%)	357 (1.8%)

nMH, non-migraine headaches; COPD, chronic obstructive pulmonary disease; GERD, gastroesophageal reflux disease; FGID, functional gastrointestinal disorder; IBS, irritable bowel syndrome.
